# The genome sequence of lesser trefoil or Irish shamrock, *Trifolium dubium* Sibth. (Fabaceae)

**DOI:** 10.12688/wellcomeopenres.21191.1

**Published:** 2024-04-24

**Authors:** Markus Ruhsam, Peter M Hollingsworth, Ann M. Mc Cartney, Katie E. Herron, Graham M. Hughes, Maarten J. M. Christenhusz, Michael F. Fay, Ilia J. Leitch, Royal Botanic Garden Edinburgh Genome Acquisition Lab, Plant Genome Sizing collective, Darwin Tree of Life Barcoding collective, Wellcome Sanger Institute Tree of Life Management, Samples and Laboratory team, Wellcome Sanger Institute Scientific Operations: Sequencing Operations, Wellcome Sanger Institute Tree of Life Core Informatics team, Tree of Life Core Informatics collective, Darwin Tree of Life Consortium

**Affiliations:** 1 Royal Botanic Garden Edinburgh, Edinburgh, Scotland, UK; 2 University of California Santa Cruz, Santa Cruz, California, USA; 3 University College Dublin, Dublin, Leinster, Ireland; 4 Royal Botanic Gardens Kew, Richmond, England, UK; 5 Curtin University, Perth, Western Australia, Australia

**Keywords:** Trifolium dubium, lesser trefoil, genome sequence, chromosomal, Fabales

## Abstract

We present a genome assembly from an individual *Trifolium dubium* (lesser trefoil; Tracheophyta; Magnoliopsida; Fabales; Fabaceae) as part of a collaboration between the Darwin Tree of Life and the European Reference Genome Atlas. The genome sequence is 679.1 megabases in span. Most of the assembly is scaffolded into 15 chromosomal pseudomolecules. The two mitochondrial genomes have lengths of 133.86 kb and 182.32 kb, and the plastid genome assembly has a length of 126.22 kilobases.

## Abstract (ga)

Cuirimid i láthair geanóm tionól ó an dhuine aonair seamróg na hÉireann mar chuid de a comhoibriú idir an Darwin Crann na Beatha agus an Atlas Géanóm Tagartha na hEorpa. Is an geanóm sheicheamhú 679.1megabases fad. An chuid is mó den tionól scafall isteach 15 crómasómach pseudomolecules. Ta an dhá mitochondrial geanóm tá faid de 133.86 kb agus 182.32 kb, agus an plastid geanóm tionól tá fad de 126.22 kb.

## Species taxonomy

Eukaryota; Viridiplantae; Streptophyta; Streptophytina; Embryophyta; Tracheophyta; Euphyllophyta; Spermatophyta; Magnoliopsida; Mesangiospermae; eudicotyledons; Gunneridae; Pentapetalae; rosids; fabids; Fabales; Fabaceae; Papilionoideae; 50 kb inversion clade; NPAAA clade; Hologalegina; IRL clade; Trifolieae; *Trifolium*; *Trifolium dubium* Sibth. (NCBI:txid97021).

## Background

Lesser trefoil ( *Trifolium dubium* Sibth.), also known as lesser hop clover or suckling clover, is a common clover species that is considered by most to represent the traditional Irish shamrock. It is native and common across Europe, north to Scandinavia and south to Morocco and Turkey, but it is also found in many temperate regions of the world as an introduced species ( POWO, 2023).

**Figure 1.**
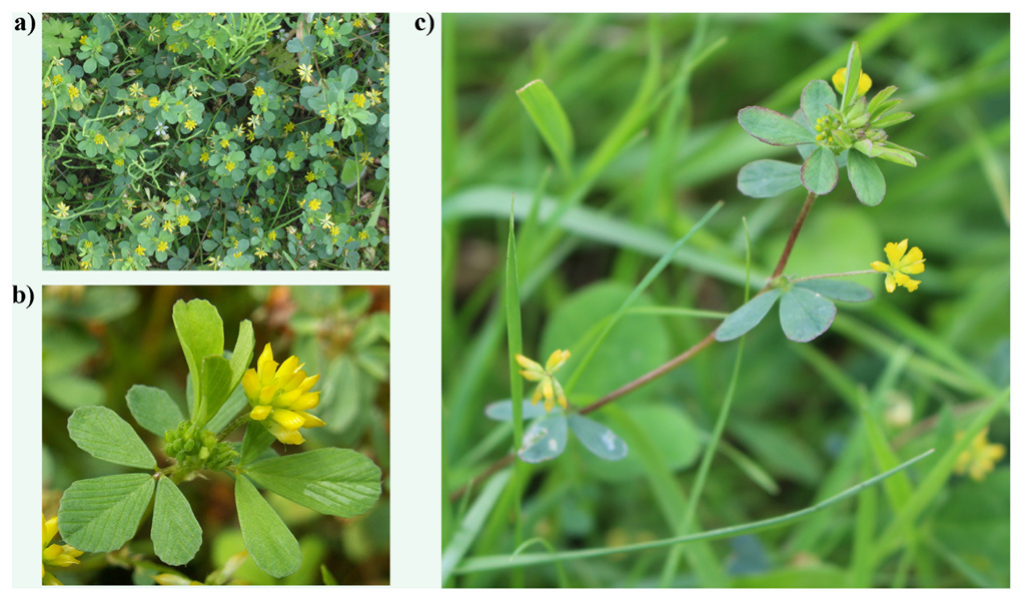
Photographs of *Trifolium dubium* ( **a** and **b** are representative images for the species, but not the specimen pr population used for genome sequencing, **c** is a representative plant from the population that was used for genome sequencing). **a**) https://commons.wikimedia.org/wiki/User:Rasbak **b**) https://commons.wikimedia.org/wiki/User:Kenraiz **c**) Markus Ruhsam.

*Trifolium dubium* is a mat-forming annual, which has up to 20 tiny yellow flowers packed in dense globular flower heads ( Figure 1). Most commonly, it occurs in unimproved grassland, but is also found in other habitats such as lawns, pastures, coastal meadows, roadsides, waste places and disturbed areas. Its adaptability to different environmental conditions has contributed to its prevalence in both natural and anthropogenic landscapes across its range.

There has been much discussion on the identity of the “true” shamrock, but for over a century the majority of people surveyed consider *T. dubium* to be the real one ( Colgan, 1892; Colgan, 1893; Nelson, 1991). Shamrock flowers from May to October in Ireland, so it is not generally in flower on St Patrick’s Day (17 March); however, leaves of *T. dubium* are worn on St. Patrick’s Day, and have since become a floral symbol of Ireland. *Trifolim dubium* appears in numerous emblems of state and non-state organisations and companies across the Republic of Ireland, Northern Ireland, and beyond. Together with the harp, the shamrock is registered as an international trademark by the Government of Ireland.

The legend of the shamrock holds that St Patrick used its three-parted clover leaflets to explain to the Irish people the Christian concept of the Holy Trinity ( Van Treeck & Croft, 1936), although the word “shamrock” derives from the Irish words *seamair* (clover) and *óg* (young) ( Nelson, 1991).

While *T. dubium* is not typically cultivated as a primary crop, like most legumes it is capable of fixing atmospheric nitrogen through its symbiotic relationship with nitrogen-fixing bacteria in root nodules ( Brock, 1973). This enriches the soil as well as the plants themselves, which therefore provide a good source of macro- and micronutrients and protein for livestock ( Brock, 1973; Gounden *et al.*, 2018). This species and several related species of *Trifolium* also produce condensed tannins (unlike the major crop clover species *T. repens* L. and *T. pratense* L.), making them of interest to breeding programmes of forage legumes, because they are less likely to cause legume bloat in ruminants ( Fay & Dale, 1993).

While many cytological studies of *Trifolium* species have indicated that most (about 80%) are diploid based on *x* = 8 (with descending dysploidy giving rise to *x* = 7, 6 or 5 in some species; Ellison *et al.*, 2006), counts of *T. dubium* have suggested it is a tetraploid, although there has been some discrepancy as to whether it is 2 *n* = 28 or 30 ( Ansari *et al.*, 2008; Taylor *et al.*, 1983; Vižintin *et al.*, 2006; Zohary & Heller, 1984), or 2 *n* = 32 (based on a chromosome count of a plant from Kent, England; Gornall and Bailey, 1993). Recent molecular cytogenetic studies of *T. dubium* with 2 *n* = 30, are in agreement with the genome assembly reported here, and have provided important insights into its genetic composition and evolution (e.g. Ansari *et al.*, 2008; Vozárová *et al.*, 2021). Such studies have proposed that the species is an allotetraploid that likely arose from natural hybridisation between *T. campestre* Schreb. (2 *n* = 14) and *T. micranthum* Viv. (2 *n* = 16) ( Ansari *et al.*, 2008).

Whole genome sequence data are now available for at least six *Trifolium* species (e.g. Bickhart *et al.*, 2022; Garg *et al.*, 2022; Griffiths *et al.*, 2019; Santangelo *et al.*, 2023), and here we present the first high-quality genome for *T. dubium*, stemming from a collaboration involving the Darwin Tree of Life Project and the European Reference Genome Atlas pilot project. We anticipate this genome will be a valuable genomic resource for a range of future studies. These include comparative analyses focused on the evolution of allopolyploid genomes, as well as studies exploring its potential as an additional nutritional source for livestock, especially given its high condensed tannin content.

## Genome sequence report

The genome was sequenced from a specimen of *Trifolium dubium* collected from Gorebridge, UK (55.84, –3.04). Using flow cytometry, the genome size (1C-value) was estimated to be 0.84 pg, equivalent to 820 Mb. A total of 72-fold coverage in Pacific Biosciences single-molecule HiFi long reads was generated. Primary assembly contigs were scaffolded with chromosome conformation Hi-C data. Manual assembly curation corrected 283 missing joins or mis-joins, reducing the scaffold number by 61.95%, and increasing the scaffold N50 by 14.41%.

**Figure 2.**
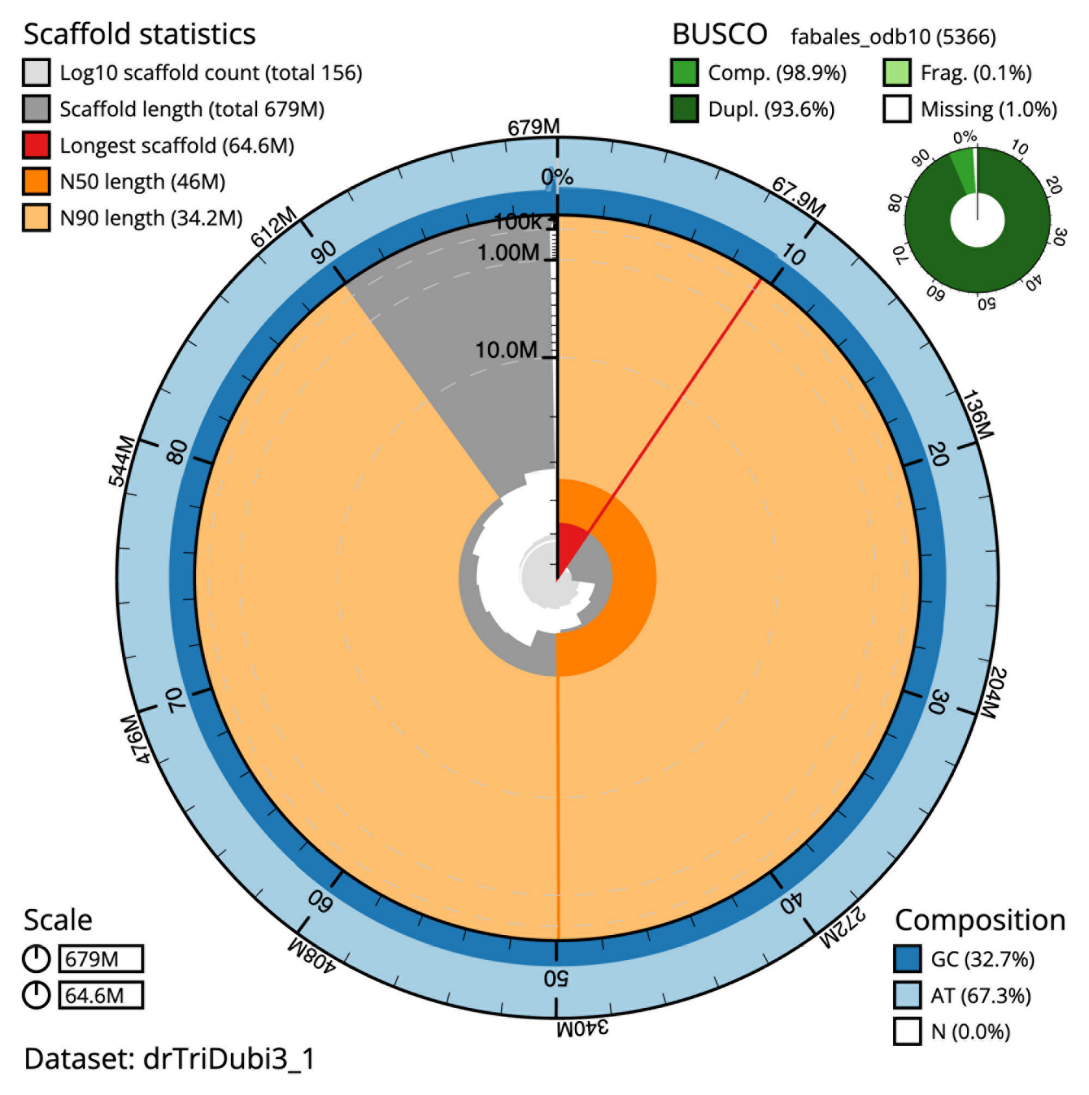
Genome assembly of *Trifolium dubium*, drTriDubi3.1: metrics. The BlobToolKit Snailplot shows N50 metrics and BUSCO gene completeness. The main plot is divided into 1,000 size-ordered bins around the circumference with each bin representing 0.1% of the 679,499,717 bp assembly. The distribution of scaffold lengths is shown in dark grey with the plot radius scaled to the longest scaffold present in the assembly (64,644,275 bp, shown in red). Orange and pale-orange arcs show the N50 and N90 scaffold lengths (46,006,535 and 34,190,264 bp), respectively. The pale grey spiral shows the cumulative scaffold count on a log scale with white scale lines showing successive orders of magnitude. The blue and pale-blue area around the outside of the plot shows the distribution of GC, AT and N percentages in the same bins as the inner plot. A summary of complete, fragmented, duplicated and missing BUSCO genes in the fabales_odb10 set is shown in the top right. An interactive version of this figure is available at https://blobtoolkit.genomehubs.org/view/drTriDubi3_1/dataset/drTriDubi3_1/snail.

**Figure 3.**
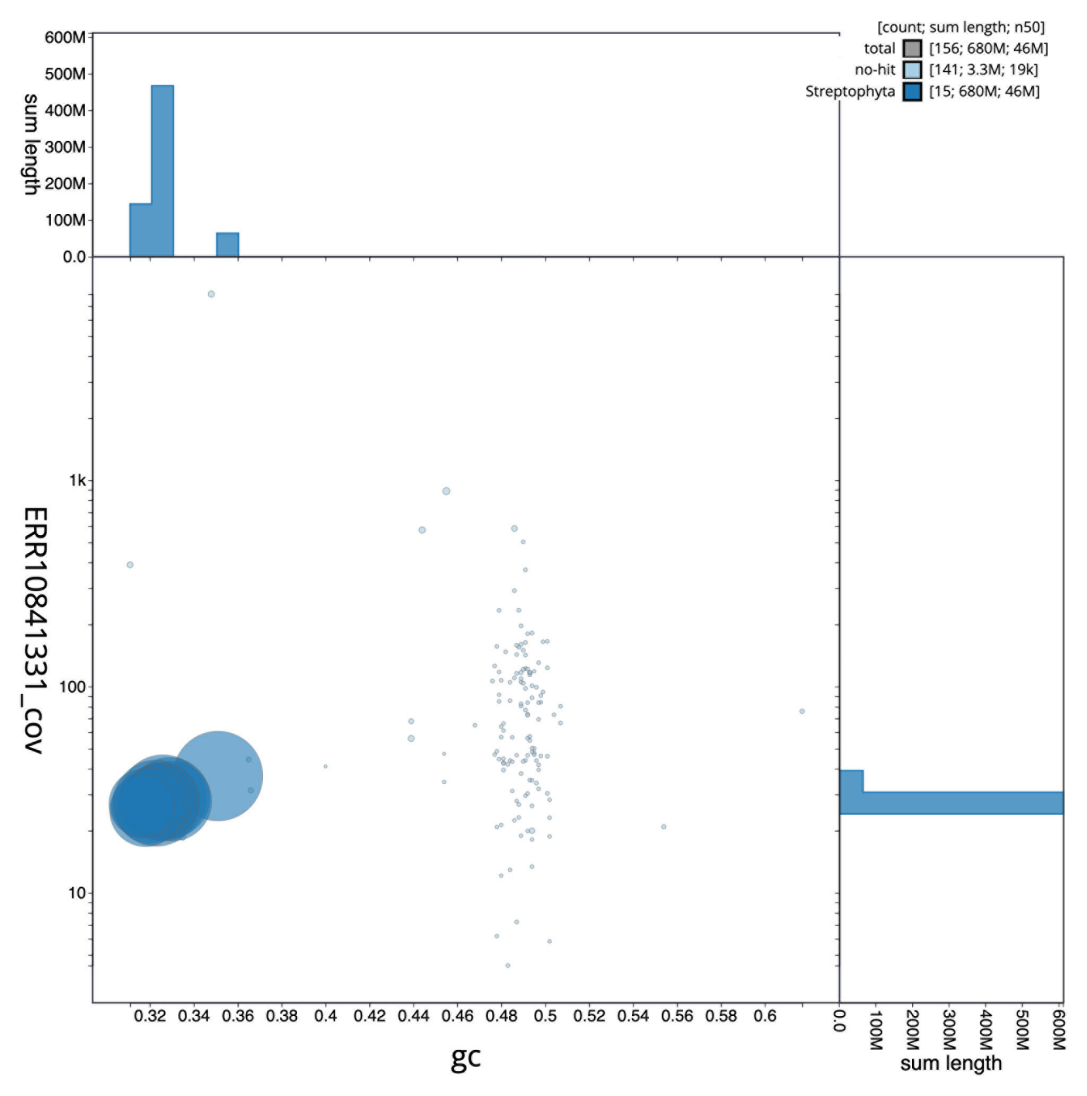
Genome assembly of *Trifolium dubium*, drTriDubi3.1: BlobToolKit GC-coverage plot. Scaffolds are coloured by phylum. Circles are sized in proportion to scaffold length. Histograms show the distribution of scaffold length sum along each axis. An interactive version of this figure is available at https://blobtoolkit.genomehubs.org/view/drTriDubi3_1/dataset/drTriDubi3_1/blob.

**Figure 4.**
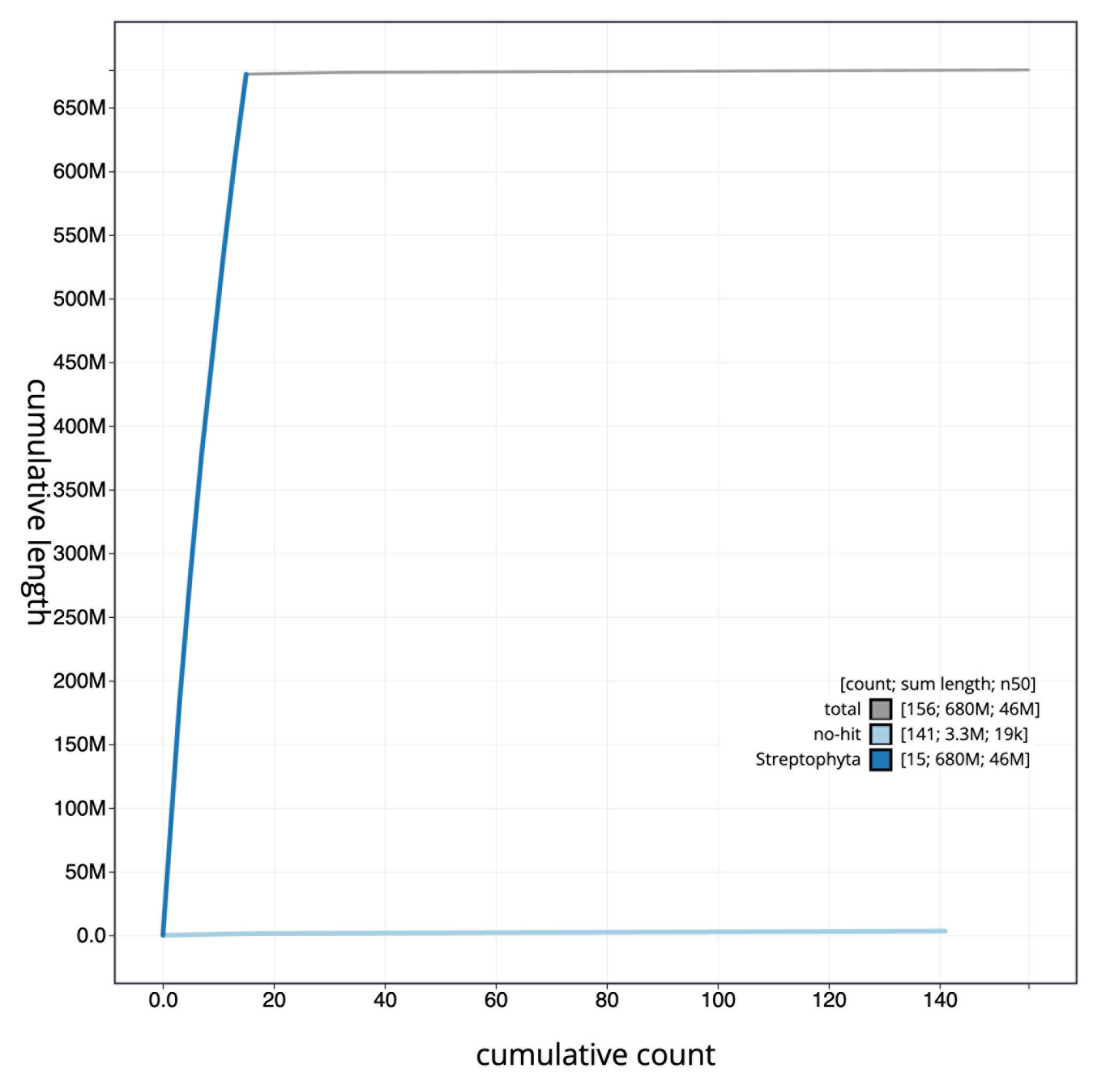
Genome assembly of *Trifolium dubium*, drTriDubi3.1: BlobToolKit cumulative sequence plot. The grey line shows cumulative length for all scaffolds. Coloured lines show cumulative lengths of scaffolds assigned to each phylum using the buscogenes taxrule. An interactive version of this figure is available at https://blobtoolkit.genomehubs.org/view/drTriDubi3_1/dataset/drTriDubi3_1/cumulative.

**Figure 5.**
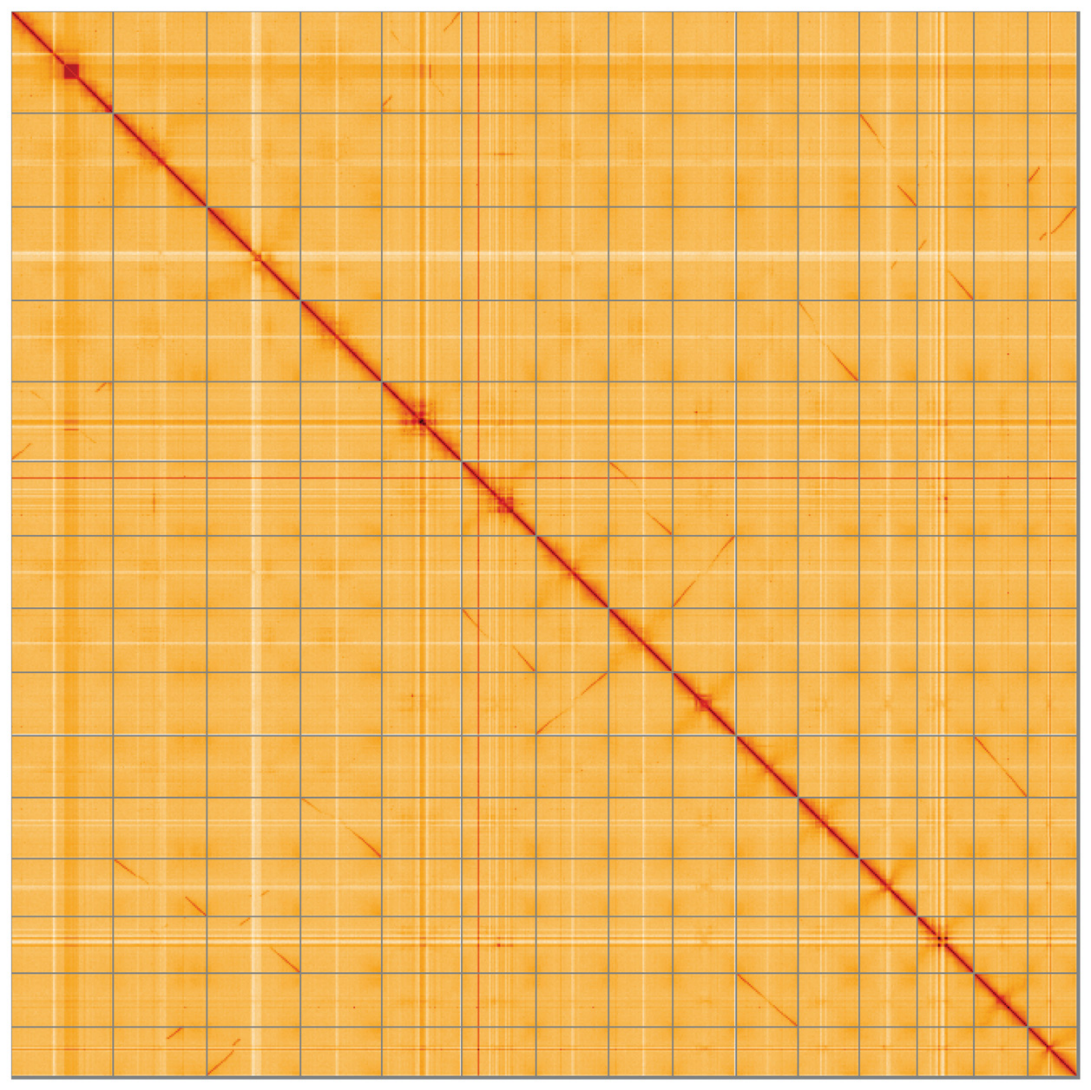
Genome assembly of *Trifolium dubium*, drTriDubi3.1: Hi-C contact map of the drTriDubi3.1 assembly, visualised using HiGlass. Chromosomes are shown in order of size from left to right and top to bottom. An interactive version of this figure may be viewed at https://genome-note-higlass.tol.sanger.ac.uk/l/?d=F0MkUMXRRMqAc9HJXoQ8LA.

The final assembly has a total length of 679.1 Mb in 153 sequence scaffolds with a scaffold N50 of 46.0 Mb ( Table 1). The snail plot in Figure 2 provides a summary of the assembly statistics, while the distribution of assembly scaffolds on GC proportion and coverage is shown in Figure 3. The cumulative assembly plot in Figure 4 shows curves for subsets of scaffolds assigned to different phyla. Most (99.51%) of the assembly sequence was assigned to 15 chromosomal-level scaffolds. Chromosome-scale scaffolds confirmed by the Hi-C data are named in order of size ( Figure 5; Table 2). The order and orientation of contigs on chromosome 1 between 37.5 Mb and 42.4 Mb is uncertain. While not fully phased, the assembly deposited is of one haplotype. Contigs corresponding to the second haplotype have also been deposited. The mitochondrial and plastid genomes were also assembled and can be found as contigs within the multifasta file of the genome submission.

**Table 1.** Genome data for *Trifolium dubium*, drTriDubi3.1.

Project accession data		
Assembly identifier	drTriDubi3.1	
Species	*Trifolium dubium*	
Specimen	drTriDubi3	
NCBI taxonomy ID	97021	
BioProject	PRJEB59394	
BioSample ID	SAMEA10983579	
Isolate information	drTriDubi3: flower and leaf (DNA sequencing) drTriDubi2: flower and leaf (Hi-C sequencing) drTriDubi4: flower and leaf (RNA sequencing)	
Assembly metrics *		*Benchmark*
Consensus quality (QV)	67.2	*≥ 50*
*k*-mer completeness	100.0%	*≥ 95%*
BUSCO **	C:98.9%[S:5.4%, D:93.6%], F:0.1%,M:1.0%,n:5,366	*C ≥ 95%*
Percentage of assembly mapped to chromosomes	99.51%	*≥ 95%*
Sex chromosomes	None	*localised homologous pairs*
Organelles	Mitochondrial genomes: 133.86 kb and 182.32 kb Plastid genome: 126.22 kb	*complete single alleles*
Raw data accessions		
PacificBiosciences SEQUEL II	ERR10841331	
Hi-C Illumina	ERR10851537	
PolyA RNA-Seq Illumina	ERR10908617	
Genome assembly		
Assembly accession	GCA_951804385.1	
*Accession of alternate haplotype*	GCA_951804395.1	
Span (Mb)	679.1	
Number of contigs	742	
Contig N50 length (Mb)	3.0	
Number of scaffolds	153	
Scaffold N50 length (Mb)	46.0	
Longest scaffold (Mb)	64.64	

* Assembly metric benchmarks are adapted from column VGP-2020 of “Table 1: Proposed standards and metrics for defining genome assembly quality” from ( Rhie *et al.*, 2021). ** BUSCO scores based on the fabales_odb10 BUSCO set using version 5.3.2. C = complete [S = single copy, D = duplicated], F = fragmented, M = missing, n = number of orthologues in comparison. A full set of BUSCO scores is available at https://blobtoolkit.genomehubs.org/view/drTriDubi3_1/dataset/drTriDubi3_1/busco.

**Table 2.** Chromosomal pseudomolecules in the genome assembly of *Trifolium dubium*, drTriDubi3.

INSDC accession	Chromosome	Length (Mb)	GC%
OX638062.1	1	64.64	35.0
OX638063.1	2	59.51	32.5
OX638064.1	3	59.37	32.5
OX638065.1	4	51.57	33.0
OX638066.1	5	50.66	33.0
OX638067.1	6	47.18	32.5
OX638068.1	7	46.01	32.5
OX638069.1	8	40.67	32.5
OX638070.1	9	40.21	32.0
OX638071.1	10	39.28	32.5
OX638072.1	11	38.74	32.0
OX638073.1	12	36.76	31.5
OX638074.1	13	35.95	32.0
OX638075.1	14	34.19	32.0
OX638076.1	15	31.43	31.5
OX638079.1	Pltd	0.13	35.0
OX638077.1	MT1	0.13	44.5
OX638078.1	MT2	0.18	45.5

The estimated Quality Value (QV) of the final assembly is 67.2 with *k*-mer completeness of 100.0%, and the assembly has a BUSCO v5.3.2 completeness of 98.9% (single = 5.4%, duplicated = 93.6%), using the fabales_odb10 reference set ( *n* = 5,366).

Metadata for specimens, barcode results, spectra estimates, sequencing runs, contaminants and pre-curation assembly statistics are given at https://tolqc.cog.sanger.ac.uk/darwin/dicots/Trifolium_dubium/.

## Methods

### Sample acquisition, genome size estimation and nucleic acid extraction

Leaf and flower samples of *Trifolium dubium* were collected from Gorebridge, Scotland, UK (latitude 55.84, longitude –3.04) on 2021-08-11. One specimen was used for DNA sequencing (specimen ID EDTOL02342, ToLID drTriDubi3); another was used for Hi-C sequencing (specimen ID EDTOL02341, ToLID drTriDubi2); and a third specimen was used for RNA sequencing (specimen ID EDTOL02343, ToLID drTriDubi4). The specimens were collected and identified by Markus Ruhsam (Royal Botanic Garden Edinburgh) and preserved in liquid nitrogen. A voucher specimen from the same population of the sequenced plant is housed in the herbarium of the Royal Botanic Garden Edinburgh (E), available at https://data.rbge.org.uk/herb/E01152325.

The genome size was estimated by flow cytometry using the fluorochrome propidium iodide and following the ‘one-step’ method as outlined in Pellicer *et al.* (2021). The General Purpose Buffer (GPB) supplemented with 3% PVP and 0.08% (v/v) beta-mercaptoethanol was used for isolation of nuclei ( Loureiro *et al.*, 2007), and the internal calibration standard was *Petroselinum crispum* ‘Champion Moss Curled’ with an assumed 1C-value of 2,200 Mb ( Obermayer *et al.*, 2002).

The workflow for high molecular weight (HMW) DNA extraction at the Wellcome Sanger Institute (WSI) includes a sequence of core procedures: sample preparation; sample homogenisation, DNA extraction, fragmentation, and clean-up. In sample preparation, the drTriDubi3 sample was weighed and dissected on dry ice ( Jay *et al.*, 2023).

For sample homogenisation, flower and leaf tissue was cryogenically disrupted using the Covaris cryoPREP ^®^ Automated Dry Pulverizer ( Narváez-Gómez *et al.*, 2023). HMW DNA was extracted using the Automated Plant MagAttract v2 protocol ( Todorovic *et al.*, 2023a). HMW DNA was sheared into an average fragment size of 12–20 kb in a Megaruptor 3 system with speed setting 30 ( Todorovic *et al.*, 2023b). Sheared DNA was purified by solid-phase reversible immobilisation ( Strickland *et al.*, 2023): in brief, the method employs a 1.8X ratio of AMPure PB beads to sample to eliminate shorter fragments and concentrate the DNA. The concentration of the sheared and purified DNA was assessed using a Nanodrop spectrophotometer and Qubit Fluorometer and Qubit dsDNA High Sensitivity Assay kit. Fragment size distribution was evaluated by running the sample on the FemtoPulse system.

RNA was extracted from flower tissue of drTriDubi4 in the Tree of Life Laboratory at the WSI using the RNA Extraction: Automated MagMax™ *mir*Vana protocol ( do Amaral *et al.*, 2023). The RNA concentration was assessed using a Nanodrop spectrophotometer and a Qubit Fluorometer using the Qubit RNA Broad-Range Assay kit. Analysis of the integrity of the RNA was done using the Agilent RNA 6000 Pico Kit and Eukaryotic Total RNA assay.

Protocols developed by the WSI Tree of Life core laboratory are publicly available on protocols.io ( Denton *et al.*, 2023).

### Sequencing

Pacific Biosciences HiFi circular consensus DNA sequencing libraries were constructed according to the manufacturers’ instructions. Poly(A) RNA-Seq libraries were constructed using the NEB Ultra II RNA Library Prep kit. DNA and RNA sequencing was performed by the Scientific Operations core at the WSI on Pacific Biosciences SEQUEL II (HiFi) and Illumina NovaSeq 6000 (RNA-Seq) instruments. Hi-C data were also generated from flower and leaf tissue of drTriDubi2 using the Arima2 kit and sequenced on the Illumina NovaSeq 6000 instrument.

### Genome assembly, curation and evaluation

Assembly was carried out with HiCanu ( Nurk *et al.*, 2020) and haplotypic duplication was identified and removed with purge_dups ( Guan *et al.*, 2020). The assembly was then scaffolded with Hi-C data ( Rao *et al.*, 2014) using YaHS ( Zhou *et al.*, 2023). The assembly was checked for contamination and corrected as described previously ( Howe *et al.*, 2021). Manual curation was performed using HiGlass ( Kerpedjiev *et al.*, 2018) and PretextView ( Harry, 2022). The organelle genomes were assembled using MitoHiFi ( Uliano-Silva *et al.*, 2023) and OATK ( Zhou, 2023).

A Hi-C map for the final assembly was produced using bwa-mem2 ( Vasimuddin *et al.*, 2019) in the Cooler file format ( Abdennur & Mirny, 2020). To assess the assembly metrics, the *k*-mer completeness and QV consensus quality values were calculated in Merqury ( Rhie *et al.*, 2020). This work was done using Nextflow ( Di Tommaso *et al.*, 2017) DSL2 pipelines “sanger-tol/readmapping” ( Surana *et al.*, 2023a) and “sanger-tol/genomenote” ( Surana *et al.*, 2023b). The genome was analysed within the BlobToolKit environment ( Challis *et al.*, 2020) and BUSCO scores ( Manni *et al.*, 2021; Simão *et al.*, 2015) were calculated.

Table 3 contains a list of relevant software tool versions and sources.

**Table 3.** Software tools: versions and sources.

Software tool	Version	Source
BlobToolKit	4.1.7	https://github.com/blobtoolkit/blobtoolkit
BUSCO	5.3.2	https://gitlab.com/ezlab/busco
HiCanu	2.2	https://github.com/marbl/canu
HiGlass	1.11.6	https://github.com/higlass/higlass
Merqury	MerquryFK	https://github.com/thegenemyers/MERQURY.FK
MitoHiFi	2	https://github.com/marcelauliano/MitoHiFi
OATK	0.1	https://github.com/c-zhou/oatk
PretextView	0.2	https://github.com/wtsi-hpag/PretextView
purge_dups	1.2.3	https://github.com/dfguan/purge_dups
sanger-tol/genomenote	v1.0	https://github.com/sanger-tol/genomenote
sanger-tol/readmapping	1.1.0	https://github.com/sanger-tol/readmapping/tree/1.1.0
YaHS	1.1a.2	https://github.com/c-zhou/yahs

### Wellcome Sanger Institute – Legal and Governance

The materials that have contributed to this genome note have been supplied by a Darwin Tree of Life Partner. The submission of materials by a Darwin Tree of Life Partner is subject to the **‘Darwin Tree of Life Project Sampling Code of Practice’**, which can be found in full on the Darwin Tree of Life website here. By agreeing with and signing up to the Sampling Code of Practice, the Darwin Tree of Life Partner agrees they will meet the legal and ethical requirements and standards set out within this document in respect of all samples acquired for, and supplied to, the Darwin Tree of Life Project. 

Further, the Wellcome Sanger Institute employs a process whereby due diligence is carried out proportionate to the nature of the materials themselves, and the circumstances under which they have been/are to be collected and provided for use. The purpose of this is to address and mitigate any potential legal and/or ethical implications of receipt and use of the materials as part of the research project, and to ensure that in doing so we align with best practice wherever possible. The overarching areas of consideration are:

• Ethical review of provenance and sourcing of the material

• Legality of collection, transfer and use (national and international) 

Each transfer of samples is further undertaken according to a Research Collaboration Agreement or Material Transfer Agreement entered into by the Darwin Tree of Life Partner, Genome Research Limited (operating as the Wellcome Sanger Institute), and in some circumstances other Darwin Tree of Life collaborators.

## Data Availability

European Nucleotide Archive: *Trifolium dubium*. Accession number PRJEB59394; https://identifiers.org/ena.embl/PRJEB59394 ( Wellcome Sanger Institute, 2023). The genome sequence is released openly for reuse. The *Trifolium dubium* genome sequencing initiative is part of the European Reference Genome Atlas Pilot Project ( https://www.erga-biodiversity.eu/pilot-project) as well as the Darwin Tree of Life (DToL) project. All raw sequence data and the assembly have been deposited in INSDC databases. The genome will be annotated using available RNA-Seq data and presented through the Ensembl pipeline at the European Bioinformatics Institute. Raw data and assembly accession identifiers are reported in Table 1.

## Author information

Members of the Royal Botanic Garden Edinburgh Genome Acquisition Lab are listed here: https://doi.org/10.5281/zenodo.4786682.

Members of the Plant Genome Sizing collective are listed here: https://doi.org/10.5281/zenodo.7994306.

Members of the Darwin Tree of Life Barcoding collective are listed here: https://doi.org/10.5281/zenodo.4893703.

Members of the Wellcome Sanger Institute Tree of Life Management, Samples and Laboratory team are listed here: https://doi.org/10.5281/zenodo.10066175.

Members of Wellcome Sanger Institute Scientific Operations: Sequencing Operations are listed here: https://doi.org/10.5281/zenodo.10043364.

Members of the Wellcome Sanger Institute Tree of Life Core Informatics team are listed here: https://doi.org/10.5281/zenodo.10066637.

Members of the Tree of Life Core Informatics collective are listed here: https://doi.org/10.5281/zenodo.5013541.

Members of the Darwin Tree of Life Consortium are listed here: https://doi.org/10.5281/zenodo.4783558.

## References

[ref-1] Abdennur N Mirny LA : Cooler: scalable storage for Hi-C data and other genomically labeled arrays. *Bioinformatics.* 2020;36(1):311–316. 10.1093/bioinformatics/btz540 31290943 PMC8205516

[ref-80] Ansari HA Ellison NW Williams WM : Molecular and cytogenetic evidence for an allotetraploid origin of *Trifolium dubium* (Leguminosae). *Chromosoma.* 2008;117(2):159–167. 10.1007/s00412-007-0134-4 18058119

[ref-56] Bickhart DM Koch LM Smith TPL : Chromosome-scale assembly of the highly heterozygous genome of red clover ( *Trifolium pratense* L.), an allogamous forage crop species. *GigaByte.* 2022;2022: gigabyte42. 10.46471/gigabyte.42 36824517 PMC9650271

[ref-57] Brock JL : Growth and nitrogen fixation of pure stands of three pasture legumes with high/low phosphate. *New Zealand Journal of Agricultural Research.* 1973;16(4):483–491. 10.1080/00288233.1973.10421093

[ref-7] Challis R Richards E Rajan J : BlobToolKit - interactive quality assessment of genome assemblies. *G3 (Bethesda).* 2020;10(4):1361–1374. 10.1534/g3.119.400908 32071071 PMC7144090

[ref-58] Colgan N : The shamrock: an attempt to fix its species. *The Irish Naturalist.* 1892;1(5):95–97. Reference Source

[ref-59] Colgan N : The shamrock: a further attempt to fix its species. *The Irish Naturalist.* 1893;2(8):207–211. Reference Source

[ref-41] Denton A Yatsenko H Jay J : Sanger Tree of Life wet laboratory protocol collection V.1. *protocols.io.* 2023. 10.17504/protocols.io.8epv5xxy6g1b/v1

[ref-10] Di Tommaso P Chatzou M Floden EW : Nextflow enables reproducible computational workflows. *Nat Biotechnol.* 2017;35(4):316–319. 10.1038/nbt.3820 28398311

[ref-42] do Amaral RJV Bates A Denton A : Sanger Tree of Life RNA extraction: automated MagMax ^TM^ mirVana. *protocols.io.* 2023. 10.17504/protocols.io.6qpvr36n3vmk/v1

[ref-60] Ellison NW Liston A Steiner JJ : Molecular phylogenetics of the clover genus ( *Trifolium*—Leguminosae). *Mol Phylogenet Evol.* 2006;39(3):688–705. 10.1016/j.ympev.2006.01.004 16483799

[ref-61] Fay MF Dale PJ : Condensed tannins in *Trifolium* species and their significance for taxonomy and plant breeding. *Genet Resour Crop Evol.* 1993;40:7–13. 10.1007/BF00053459

[ref-62] Garg V Dudchenko O Wang J : Chromosome-length genome assemblies of six legume species provide insights into genome organization, evolution, and agronomic traits for crop improvement. *J Adv Res.* 2022;42:315–329. 10.1016/j.jare.2021.10.009 36513421 PMC9788938

[ref-63] Gornall RJ Bailey JP : Cytological catalogue of the British and Irish flora botanical society of Britain and Ireland. 1993; [Date accessed 31/01/2024]. Reference Source

[ref-64] Gounden T Moodley R Jonnalagadda SB : Elemental analysis and nutritional value of edible *Trifolium* (clover) species. *J Environ Sci Health B.* 2018;53(8):487–492. 10.1080/03601234.2018.1462923 29708825

[ref-65] Griffiths AG Moraga R Tausen M : Breaking Free: the genomics of allopolyploidy-facilitated niche expansion in white clover. *Plant Cell.* 2019;31(7):1466–1487. 10.1105/tpc.18.00606 31023841 PMC6635854

[ref-13] Guan D McCarthy SA Wood J : Identifying and removing haplotypic duplication in primary genome assemblies. *Bioinformatics.* 2020;36(9):2896–2898. 10.1093/bioinformatics/btaa025 31971576 PMC7203741

[ref-14] Harry E : PretextView (Paired REad TEXTure Viewer): a desktop application for viewing pretext contact maps.2022; [Accessed 19 October 2022]. Reference Source

[ref-15] Howe K Chow W Collins J : Significantly improving the quality of genome assemblies through curation. *GigaScience.* Oxford University Press,2021;10(1): giaa153. 10.1093/gigascience/giaa153 33420778 PMC7794651

[ref-44] Jay J Yatsenko H Narváez-Gómez JP : Sanger Tree of Life Sample preparation: triage and dissection. *protocols.io.* 2023. 10.17504/protocols.io.x54v9prmqg3e/v1

[ref-17] Kerpedjiev P Abdennur N Lekschas F : HiGlass: web-based visual exploration and analysis of genome interaction maps. *Genome Biol.* 2018;19(1): 125. 10.1186/s13059-018-1486-1 30143029 PMC6109259

[ref-66] Loureiro J Rodriguez E Dolezel J : Two new nuclear isolation buffers for plant DNA flow cytometry: a test with 37 species. *Ann Bot.* 2007;100(4):875–888. 10.1093/aob/mcm152 17684025 PMC2749623

[ref-19] Manni M Berkeley MR Seppey M : BUSCO update: novel and streamlined workflows along with broader and deeper phylogenetic coverage for scoring of eukaryotic, prokaryotic, and viral genomes. *Mol Biol Evol.* 2021;38(10):4647–4654. 10.1093/molbev/msab199 34320186 PMC8476166

[ref-46] Narváez-Gómez JP Mbye H Oatley G : Sanger Tree of Life sample homogenisation: covaris cryoPREP ^®^ automated dry pulverizer V.2. *protocols.io.* 2023. 10.17504/protocols.io.eq2lyjp5qlx9/v2

[ref-67] Nelson EC : Shamrock: botany and history of an Irish myth.Kilkenny: Boethius Press,1991. Reference Source

[ref-68] Nurk S Walenz BP Rhie A : HiCanu: accurate assembly of segmental duplications, satellites, and allelic variants from high-fidelity long reads. *Genome Res.* 2020;30(9):1291–1305. 10.1101/gr.263566.120 32801147 PMC7545148

[ref-69] Obermayer R Leitch IJ Hanson L : Nuclear DNA C-values in 30 species double the familial representation in pteridophytes. *Ann Bot.* 2002;90(2):209–217. 10.1093/aob/mcf167 12197518 PMC4240412

[ref-70] Pellicer J Powell RF Leitch IJ : The application of flow cytometry for estimating genome size, ploidy level endopolyploidy, and reproductive modes in plants.In: Besse, P. (ed.) *Methods Mol Biol.* New York, NY: Humana,2021;2222:325–361. 10.1007/978-1-0716-0997-2_17 33301101

[ref-71] POWO: Plants of the world online.Royal Botanic Gardens, Kew,2023. Reference Source

[ref-47] Rao SSP Huntley MH Durand NC : A 3D map of the human genome at kilobase resolution reveals principles of chromatin looping. *Cell.* 2014;159(7):1665–1680. 10.1016/j.cell.2014.11.021 25497547 PMC5635824

[ref-22] Rhie A McCarthy SA Fedrigo O : Towards complete and error-free genome assemblies of all vertebrate species. *Nature.* 2021;592(7856):737–746. 10.1038/s41586-021-03451-0 33911273 PMC8081667

[ref-23] Rhie A Walenz BP Koren S : Merqury: reference-free quality, completeness, and phasing assessment for genome assemblies. *Genome Biol.* 2020;21(1): 245. 10.1186/s13059-020-02134-9 32928274 PMC7488777

[ref-72] Santangelo JS Battlay P Hendrickson BT : Haplotype-resolved, chromosome-level assembly of white clover ( *Trifolium repens* L., Fabaceae). *Genome Biol Evol.* 2023;15(8): evad146. 10.1093/gbe/evad146 37542471 PMC10433932

[ref-24] Simão FA Waterhouse RM Ioannidis P : BUSCO: assessing genome assembly and annotation completeness with single-copy orthologs. *Bioinformatics.* 2015;31(19):3210–3212. 10.1093/bioinformatics/btv351 26059717

[ref-49] Strickland M Cornwell C Howard C : Sanger Tree of Life fragmented DNA clean up: manual SPRI. *protocols.io.* 2023. 10.17504/protocols.io.kxygx3y1dg8j/v1

[ref-29] Surana P Muffato M Qi G : sanger-tol/readmapping: sanger-tol/readmapping v1.1.0 - Hebridean Black (1.1.0). *Zenodo.* 2023a. 10.5281/zenodo.7755669

[ref-30] Surana P Muffato M Sadasivan Baby C : sanger-tol/genomenote (v1.0.dev). *Zenodo.* 2023b. 10.5281/zenodo.6785935

[ref-73] Taylor NL Gillett JM Giri N : Morphological observations and chromosome numbers in *Trifolium* L, section *Chronosemium Ser*. *Cytologica.* 1983;48:671–677. Reference Source

[ref-74] Todorovic M Oatley G Howard C : Sanger Tree of Life HMW DNA extraction: automated plant MagAttract v.2. *protocols.io.* 2023a. 10.17504/protocols.io.36wgq3n13lk5/v1

[ref-50] Todorovic M Sampaio F Howard C : Sanger Tree of Life HMW DNA fragmentation: Diagenode Megaruptor ^®^3 for PacBio HiFi. *protocols.io.* 2023b. 10.17504/protocols.io.8epv5x2zjg1b/v1

[ref-52] Uliano-Silva M Ferreira JGRN Krasheninnikova K : MitoHiFi: a python pipeline for mitochondrial genome assembly from PacBio high fidelity reads. *BMC Bioinformatics.* 2023;24(1): 288. 10.1186/s12859-023-05385-y 37464285 PMC10354987

[ref-75] Van Treeck C Croft A : Symbols in the church.Milwaukee: Bruce Publishing Company,1936.

[ref-33] Vasimuddin M Misra S Li H : Efficient architecture-aware acceleration of BWA-MEM for multicore systems. In: *2019 IEEE International Parallel and Distributed Processing Symposium (IPDPS).*IEEE,2019;314–324. 10.1109/IPDPS.2019.00041

[ref-76] Vižintin L Javornik B Bohanec B : Genetic characterization of selected *Trifolium* species as revealed by nuclear DNA content and ITS rDNA region analysis. *Plant Science.* 2006;170(4):859–866. 10.1016/j.plantsci.2005.12.007

[ref-77] Vozárová R Macková E Vlk D : Variation in Ribosomal DNA in the Genus *Trifolium* (Fabaceae). *Plants (Basel).* 2021;10(9): 1771. 10.3390/plants10091771 34579303 PMC8465422

[ref-34] Wellcome Sanger Institute: The genome sequence of lesser trefoil or Irish shamrock, *Trifolium dubium* Sibth. (Fabaceae). European Nucleotide Archive,[dataset], accession number PRJEB59394,2023

[ref-78] Zhou C : c-zhou/oatk: Oatk-0.1. 2023. 10.5281/zenodo.7631375

[ref-53] Zhou C McCarthy SA Durbin R : YaHS: yet another Hi-C scaffolding tool. *Bioinformatics.* 2023;39(1): btac808. 10.1093/bioinformatics/btac808 36525368 PMC9848053

[ref-79] Zohary M Heller D : The genus *Trifolium*.Jerusalem: Israel Academy of Sciences and Humanities,1984. Reference Source

