# Supplementary material for: The genome sequence of lesser trefoil or Irish shamrock, Trifolium dubium Sibth. (Fabaceae)
Source: Wellcome Open Res. 2024 Apr 24;9:229. [Version 1] doi: 10.12688/wellcomeopenres.21191.1 (PMC11384199; doi:10.12688/wellcomeopenres.21191.1)
Supplement: Peer review history

# Reviewer report: Reviewer response for version 1

**DOI:** 10.21956/wellcomeopenres.23438.r89306

**Published:** 2024-09-09

**Authors:** Yoshinori Fukasawa

**Affiliations:** 1 Center for Center for Bioscience Research and Education, Utsunomiya University, Tochigi, Japan

This manuscript reports the genome sequence of Trifolium dubium, the Irish Shamrock, assembled from PacBio HiFi and Hi-C data. The final assembly (drTriDubi3.1) has a total length of 679.1 Mb in 153 sequence scaffolds, with a scaffold N50 of 46.0 Mb and 99.51% of the assembled sequence assigned to 15 chromosomal-level scaffolds. The authors also report the assembly of the mitochondrial (133.86 kb and 182.32 kb) and plastid (126.22 kb) genomes.

**Major Points:**

 - **Haplotype phasing:** The manuscript states that the assembly represents one haplotype and that contigs from the second haplotype were also deposited. However, the statistics for the phased assembly are not available. It would be beneficial to clarify this aspect and explain the rationale behind not aiming for a fully phased assembly.

 - **Chromosome 1 structure:** The manuscript indicates that the order and orientation of contigs on chromosome 1 between 37.5 Mb and 42.4 Mb are uncertain. It would be beneficial to provide further detail on the difficulties encountered in this region and to present potential solutions or avenues for further investigation that could be pursued in order to resolve this ambiguity.

 - **Genome annotation: **The manuscript makes a cursory mention of the planned use of RNA-Seq data for genome annotation and presentation through Ensemble. However, providing a more detailed rationale for the decision to extract RNA only from flower tissue would be beneficial for readers. Additionally, discussing the expected timeline for the annotation release would be helpful for researchers interested in utilizing this resource. 

**Minor Points:**

 - It is imperative that the source of the k-mers used in Merqury be explicitly described. This issue could be addressed by specifying whether the k-mers are derived from HiFi reads. If this is the case, readers could consider that this could potentially lead to an overestimation of QV within the Merqury framework.

 - Subgenome identification: The authors may employ existing genomic resources of the proposed parental species (T. campestre and T. micranthum) to identify homeologous regions within the T. dubium assembly.

Are sufficient details of methods and materials provided to allow replication by others?

Yes

Is the rationale for creating the dataset(s) clearly described?

Yes

Are the datasets clearly presented in a useable and accessible format?

Yes

Are the protocols appropriate and is the work technically sound?

Yes

Reviewer Expertise:

Plant genomics / bioinformatics

I confirm that I have read this submission and believe that I have an appropriate level of expertise to confirm that it is of an acceptable scientific standard.

# Reviewer report: Reviewer response for version 1

**DOI:** 10.21956/wellcomeopenres.23438.r87955

**Published:** 2024-08-26

**Authors:** Rizky Dwi Satrio

**Affiliations:** 1 Department of Biology, The Republic of Indonesia Defense University, Bogor, Indonesia; 2 Universitas Pertahanan Indonesia, Tajur, Indonesia

The manuscript titled "The genome sequence of lesser trefoil or Irish shamrock, *Trifolium dubium* Sibth. (Fabaceae)" presents a high-quality genome assembly of *Trifolium dubium*, a species often considered to represent the traditional Irish shamrock. The genome was sequenced as part of a collaboration between the Darwin Tree of Life and the European Reference Genome Atlas. The study reports a genome assembly size of 679.1 megabases, scaffolded into 15 chromosomal pseudomolecules, along with two mitochondrial genomes and a plastid genome.

**Review**

*Rationale for Creating the Dataset*

- Clarity: The manuscript clearly explains the motivation behind sequencing the *Trifolium dubium* genome. The species holds cultural significance and has potential agricultural importance due to its ability to fix nitrogen and produce condensed tannins, making it a valuable resource for future comparative studies and breeding programs.
- Critique: The rationale is well articulated. However, the manuscript could benefit from a more detailed discussion of specific research questions that the dataset could address, particularly in comparative genomics and plant breeding, although this manuscript is a data note article.

*Appropriateness of Protocols and Technical Soundness*

- Protocols: The manuscript describes the sequencing and assembly methods in great detail. High molecular weight (HMW) DNA extraction was performed, followed by sequencing with PacBio HiFi, Hi-C, and RNA-Seq technologies. The assembly was curated using multiple tools, ensuring high quality.
- Technical Soundness: The work is technically sound, with a high degree of thoroughness in the methods described. The genome assembly metrics, such as scaffold N50 and BUSCO scores, indicate a high-quality assembly.
- Critique: While the protocols are appropriate and robust, it would be beneficial to include more details on the challenges faced during the assembly process and how they were addressed, especially during manual curation.

*Detailing of Methods and Materials for Replication*

- Detailing: The manuscript provides detailed descriptions of the methods, including specific protocols for DNA and RNA extraction, sequencing, and data processing. It references publicly available protocols on platforms like protocols.io, which enhances reproducibility.
- Critique: The level of detail is generally sufficient for replication. However, the manuscript could improve by providing more insights into the specific conditions used during sequencing and assembly, such as environmental factors during sample collection, particularly for RNA-seq analysis.

*Presentation and Accessibility of Datasets*

- Presentation: The datasets are presented clearly, with comprehensive tables summarizing the assembly metrics, accession numbers, and scaffold information. The manuscript also provides links to interactive tools for further exploration of the data.
- Accessibility: The data is openly accessible via public repositories, with clear instructions on how to access it.
- Critique: The presentation is strong, but the manuscript could enhance usability by providing more user-friendly summaries or visualizations, such as a Circos diagram representing an overview figure of the genome assembly for each chromosome.

Are sufficient details of methods and materials provided to allow replication by others?

Yes

Is the rationale for creating the dataset(s) clearly described?

Yes

Are the datasets clearly presented in a useable and accessible format?

Yes

Are the protocols appropriate and is the work technically sound?

Yes

Reviewer Expertise:

Plant Genomics

I confirm that I have read this submission and believe that I have an appropriate level of expertise to confirm that it is of an acceptable scientific standard.
